# Hype or hope? High placebo response in major depression treatment with ketamine and esketamine: a systematic review and meta-analysis

**DOI:** 10.3389/fpsyt.2024.1346697

**Published:** 2024-03-08

**Authors:** Alexandros Matsingos, Marcel Wilhelm, Laila Noor, Cüneyt Yildiz, Winfried Rief, Stefan G. Hofmann, Irina Falkenberg, Tilo Kircher

**Affiliations:** ^1^ Department of Psychiatry and Psychotherapy, Philipps-University Marburg, Marburg, Germany; ^2^ Department of Clinical Psychology and Psychotherapy, Philipps-University Marburg, Marburg, Germany; ^3^ Translational Clinical Psychology, Department of Psychology, Philipps-University Marburg, Marburg, Germany

**Keywords:** ketamine, esketamine, placebo, placebo response, psychoactive medication, depression (MDD), treatment expectation, NMDA-receptor antagonist

## Abstract

**Background:**

Ketamine and esketamine offer a novel approach in the pharmacological treatment of major depressive disorder (MDD). This meta-analysis aimed to investigate the placebo response in double-blind, randomized controlled studies (RCTs) on patients with MDD receiving ketamine or esketamine.

**Methods:**

For this systematic review and meta-analysis Medline (PubMed), Cochrane Central Register of Controlled Trials (CENTRAL), PsycInfo and Embase databases were systematically searched for citations published up to March 17, 2023. A total number of 5017 abstracts was identified. Quality of the included trials was assessed with the Cochrane risk-of-bias tool. The meta-analysis was performed using a restricted maximum likelihood model. This study is registered with PROSPERO, number CRD42022377591.

**Results:**

A total number of 14 studies and 1100 participants (593 in the medication group and 507 in the placebo group) meeting the inclusion criteria were selected. We estimated the pooled effect sizes of the overall placebo (*d*_pl_ = -1.85 [CI 95%: -2.9 to -0.79] and overall treatment (d_tr_ = -2.57; [CI 95% -3.36 to -1.78]) response. The overall placebo response accounts for up to 72% of the overall treatment response. Furthermore, we performed subgroup analysis of 8 studies for the for the 7 days post-intervention timepoint. Seven days post-intervention the placebo response (*d*_pl 7d_ = -1.98 [CI 95%: -3.26 to -0.69]) accounts for 66% of the treatment response (*d*_tr 7d_ = - 3.01 [CI 95%, -4.28 to -1.74]).

**Conclusion:**

Ketamine and esketamine show large antidepressant effects. However, our findings suggest that the placebo response plays a significant role in the antidepressant response and should be used for the benefit of the patients in clinical practice.

**Systematic review registration:**

https://www.crd.york.ac.uk/prospero/, identifier CRD42022377591.

## Introduction

Placebo response is one of the mechanisms contributing to treatment response of antidepressant medication. Studies have estimated that placebo response may account for up to 62-82% of treatment response in randomized controlled trials (RCTs) of oral antidepressants (1–4). A recent meta-analysis suggests that only about 15% of participants in double-blind RCTs of antidepressants may benefit from antidepressants beyond the placebo response (5). Some authors argue there is an urgent need to develop new study designs controlling for the placebo effect (6).

Ketamine and esketamine offer a novel approach in the psychopharmacological treatment of major depressive disorder (MDD). A growing number of meta-analysis indicate that NMNDA-receptor-antagonists lead to a fast reduction of depressive symptoms within hours (7–9). The US Food and Drug Administration (FDA) (10) and European Medicines Agency (EMA) (11) have authorized the use of esketamine nasal spray for the treatment of depression. Although very promising, concerns have been raised about the long-term efficacy, safety and tolerability of esketamine (12–14).

MDD is a highly relevant disease worldwide. According to the World Health Organization (WHO) 300 million people globally are affected by depression (15). MDD is the third leading cause of years lost due to disability worldwide (16). Furthermore, the consumption of antidepressant medication reached a more than double fold increase in OECD countries between 2000 and 2019 (17). However, although pharmacological treatment is well established among treatment of MDD, there are indications that only one third of patients respond to first line treatment (18). New treatment approaches and further understanding of the mechanisms underlining the antidepressant treatment response are needed in order to provide improved healthcare to patients suffering from MDD.

The extent of the placebo response in the treatment of MDD with ketamine and esketamine has not been systematically studied yet. This meta-analysis aims to estimate the placebo response in double-blind, placebo-controlled, RCTs investigating the antidepressant pharmacological treatment with ketamine or esketamine in patients with MDD.

## Methods

We conducted a systematic review and meta-analysis in accordance to the Preferred Reporting Items for Systematic Reviews and Meta-analyses (PRISMA) reporting guideline (19). The study protocol has been registered with PROSPERO number, CRD4202237759.

We included double-blinded, randomized, placebo-controlled trials with an inert placebo (e.g. saline or NaCL- infusions) as a comparator group investigating the intervention of ketamine or esketamine in a subanesthetic dose for the treatment of patients with MDD. We opted for inert placebos as a comparator group in order to avoid possible confounding pharmacological effects of active placebos (e.g. midazolam) and thus to calculate a representative placebo response in our analysis. Moreover, we excluded studies with cross-over design, active placebo, no placebo arm or no randomized controlled study design or including patients suffering of depressive symptoms due to another psychiatric condition than MDD.

We systematically searched the databases Embase, MEDLINE (PubMed), PsycINFO and the Cochrane Central Register of Controlled Trials (CENTRAL) for the keywords: placebo, esketamin* or ketamin* and depress*. A detailed overview of the search strategies for every database is provided in the supplement (**Supplementary eTable 1**). We performed the final systematic literature search on August 03, 2022. Additionally, a supplementary manual search using Google Scholar and the above-mentioned databases followed for articles published in 2023 on March 17, 2023 to identify newly published articles.

We exported results from the searches conducted at different databases and uploaded them in Rayyan, a web-based application for collaborative citation screening and full–text selection (20). After duplicate removal two independent reviewers (A.M. and L.N.) screened the abstracts and articles titles for eligibility. Following the completed eligibility check, the two reviewers screened the remaining studies for meeting the inclusion or exclusion criteria. Any discrepancies were resolved by a third reviewer (M.W.). Furthermore, we identified multiple reports of the same study and included only the report containing the information most relevant to answering the review question. Moreover, we identified study protocols of ongoing studies meeting the inclusion criteria and checked during the data extraction phase for study completion.

Data from identified reports was extracted independently by P.N. and C.T in separate uniform Microsoft Excel (21) spread sheets and compared after completion. We coded the extracted data in accordance to the guidelines provided by the Cochrane Handbook of Systematic Reviews of Interventions (22). Data extraction was supervised by A.M. We contacted authors in order to provide missing data and extracted additional data from figures using the WebPlotDigitizer tool (23) or from the study protocols of the included reports. Two independent reviewers (A.M. and L.N.) assessed study quality independently using the Cochrane risk-of-bias tool for randomized trials (RoB 2) (24). Discrepancies were resolved by a third reviewer (M.W.).

We performed the meta-analysis using the statistics software JASP (25). Missing standard deviations (SDs) were either calculated from other measures of variability (e.g. 95% confidence intervals) and test statistics or imputed from the SDs of the other similar studies (26). Placebo and treatment response were defined as the change of depressive symptoms in the placebo and medication groups respectively from baseline to the post-intervention time point. We used Cohen’s *d* effect size in a confidence interval of 95% as a measurement of effect to estimate the placebo and treatment response. A script using Pandas Python library (27) was developed in order to calculate automatically the effect sizes from the extracted data and increase precision.

Heterogeneity of the included studies was assessed by performing an *I*²-test (28). The restricted maximum-likelihood (REML) model was implemented to estimate the pooled effect size. We opted for the REML model due to its favorable outcome in comparison with other heterogeneity variance estimators (29). An exploratory univariate meta-regression analyses was performed to estimate possible moderators of the placebo or treatment response and values were considered significant as *p* <.05. We estimated the placebo and treatment response by calculating the pooled effect size for the change in depression rating scores from baseline to post-intervention in the placebo and medication groups respectively.

In order to avoid an overestimation of the placebo response we preferred the 24-hour post-intervention time point for statistical synthesis, whenever data was available, because this time point is affiliated with the highest antidepressant treatment response in ketamine and esketamine studies (30). When data for the preferred time point was not available in the selected studies, the closest available time point to the 24-hours post-intervention time point was selected. A meta-regression analysis was performed to control for the different included time points in the pooled analysis. Subgroup analysis were performed to estimate the placebo and treatment response from available data for the 40-minute, 2-hour, 4-hours, 24-hours and 7-days post-intervention time points and separately for studies with ketamine and studies with esketamine.

In order to quantify the effects of medication on study blinding we examined reported adverse events (AEs) of the included studies. We categorized reported AEs in specific and unspecific AEs. The specific AEs included medication-related AEs as described in the information leaflet for ketamine (31) and esketamine (32) and the rest of the AEs were categorized as unspecific. We formed the ratio between medication-related AEs and total AEs for each group and compared them via an unpaired t-Test to control for the validity of the categorization. Furthermore, we grouped together a subset of specific AEs containing distinct psychomimetic AEs attributed to ketamine and esketamine, like dissociation and hallucinations, that could lead to insufficient blinding. We formed the ratio between psychomimetic AEs and total AEs for each group and compared them via an unpaired t-Test to examine if they occur significantly more often in the medication arm and could be an indicator for potential bias due to insufficient blinding. More information about the categorization of the reported AEs is provided in (**Table 1**). Information about the summary of AEs in the placebo (**Supplementary eTable 2**) and medication (**Supplementary eTable 3**) group as well as a detailed report of the included non-psychomimetic and psychomimetic AEs for the placebo groups (**Supplementary eTables 4**, **5**) and for the medication groups (**Supplementary eTables 6**, **7**) is provided in the adverse events section of the supplement.

**Table 1:** AE synthesis and variable description for specific AEs.

AE synthesis and variable description for specific AEs			
Psychomimetic AEs		Non-psychomimetic AEs	
Category^1^	Symptoms^2^	Category	Symptoms
**Psychiatric symptoms**	visual hallucinations, tactile hallucinations, nightmares, paranoia, restlessness, feeling drunk, mental impairment	**Central nervous system (CNS) symptoms**	vertigo, dizziness, sedation, somnolence
**Dissociation**	dissociation, dissociation symptoms, dissociation disorder, dissociative disorder, derealization disorder	**Distorted sight**	blurred vision, altered vision depth, diplopia
**Feeling abnormal**	Feeling abnormal	**Gastrointestinal symptoms**	Nausea, vomiting
		**Sensory symptoms**	paresthesia, paresthesia oral, hypoesthesia, hypoesthesia oral

^1^Categories were formed in accordance to the information leaflet of ketamine and esketamine (31, 32).

^2^Symptoms were reported as described in the published articles or the AE section of the study protocols, thus similar symptom description like for instance dissociation and dissociation symptoms may occur.

## Results

**Figure 1:**
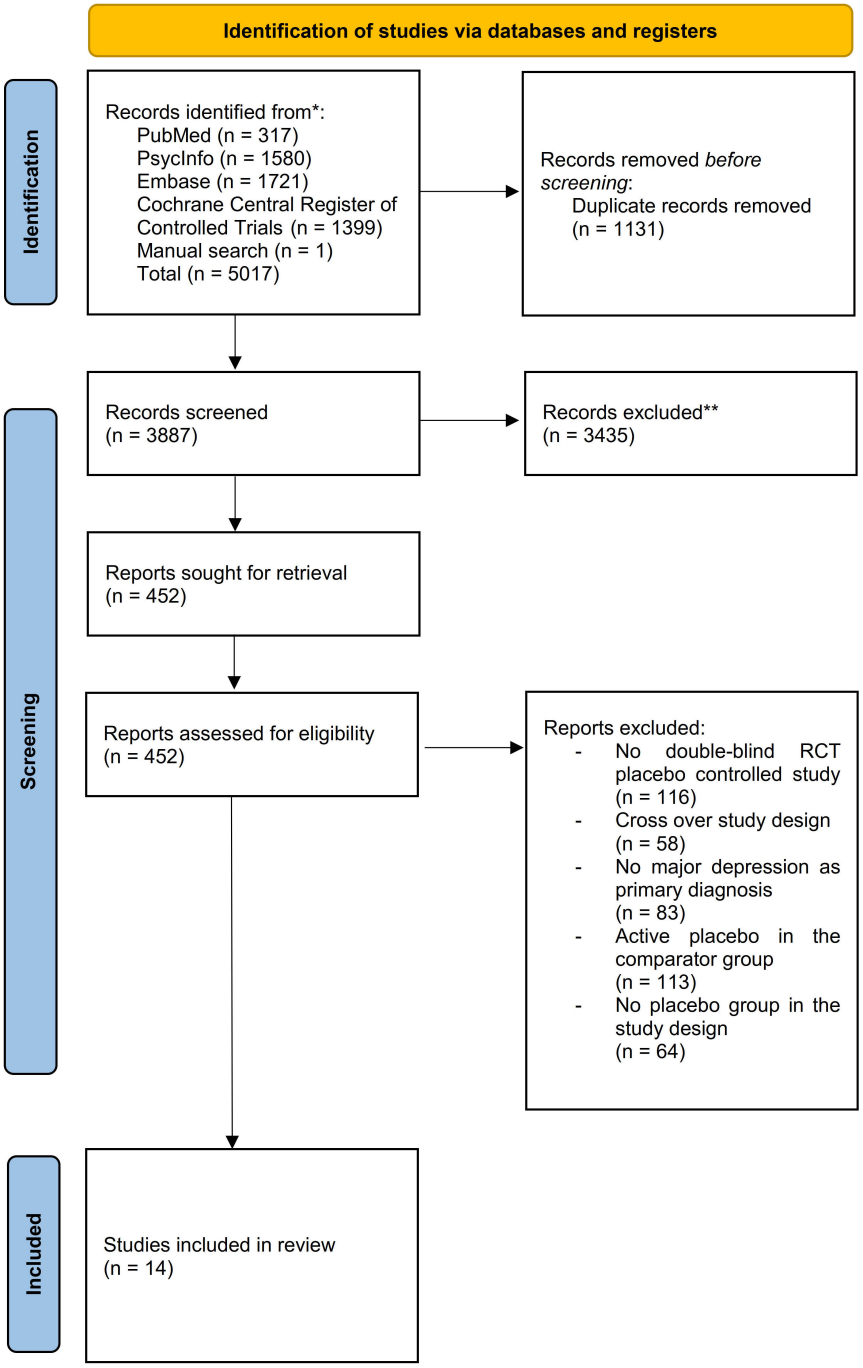
PRISMA flow diagram.

**Figure 2:**
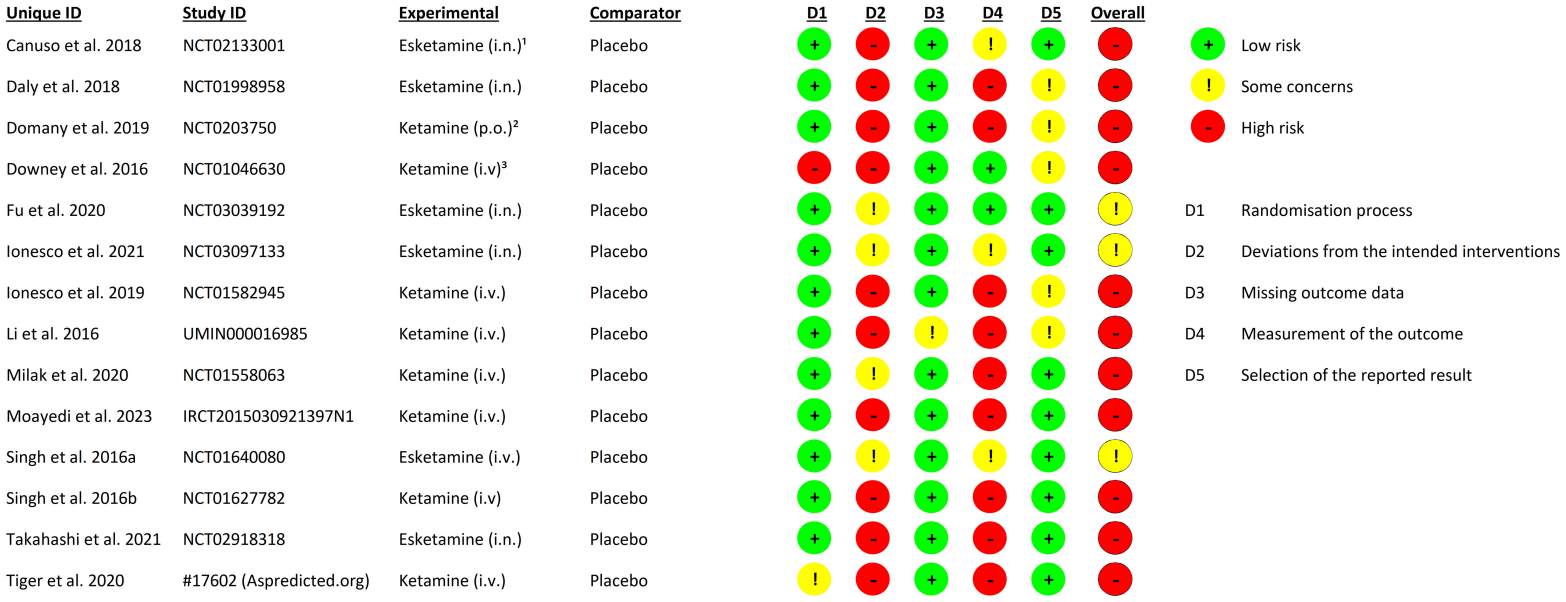
Risk of bias. ^1^ i.n., intranasal; ² p.o., per oral; ³ i.v., intravenous.

We screened 3887 abstracts that resulted from our search; assessed 452 reports for eligibility and included 14 studies (33–46) (N= 1100) meeting the inclusion criteria (**Figure 1**). The mean age of the participants was 40.09 years (SD = ± 12.75 years);57.00% were female; 593 were allocated in the medication group and 507 in the placebo group (**Table 2**). The risk of bias estimation showed 11 studies with a high risk of bias and 3 with some concerns about possible risk of bias (**Figure 2**).

**Table 2:** Study overview.

	Demographic data			Study characteristics				
Source	N	Women (% N)	Age mean (SD)	Scale	Study description	Medication^1^ (N)	Route of application^2^	Dose^3^
**Canuso et al. 2018** (33)	66	43 (65.15%)	35.8 (13.03)	MADRS	This double-blind, randomized, multicenter study compared the efficacy of intranasal esketamine or placebo for rapid reduction of depression symptoms, including suicidality, among patients with MDD at imminent suicide risk. Patients received treatment twice weekly for 4 weeks additionally to standard-of-care treatment. Depression symptoms were assessed at baseline, 4h, 24, 3d, 4d, 48h, 8d later.	ESK (35)	i.n.	84 mg
**Daly et al. 2018** (34)	67	38 (56.71%)	44.7 (10.0)	MADRS	This double-blind, randomized multicenter study examined the change in depression symptoms in MDD patient receiving placebo or one of the three different doses of intranasal esketamine (28mg, 56mg or 84mg). Depression symptoms were assessed at baseline, 2h, 24h, 7d, 14d later.	ESK (34)	i.n.	28 mg 56 mg 84 mg
**Domany et al. 2019** (35)	40	15 (37.50%)	38.3 (13.20)	MADRS	This double-blind, randomized study evaluated efficacy, safety and feasibility of repeated oral ketamine compared to placebo for outpatients with treatment-resistant MDD. Patients receive ketamine or placebo thrice weekly for three weeks. Depression symptoms were assessed at baseline, 40min, 4h, 3d, 7d and 21d later.	KET (22)	p.o.	1 mg/kg
**Downey et al.^4^ 2016** (36)	40	24 (60.00%)	27 (n/a)	MADRS BDI	In this bicenter double-blind study, patients with MDD were randomized to receive a single intravenous infusion of lanicemine, ketamine or placebo during pharmacological magnetic resonance imaging (phMRI) scan. The main objective of the study was to investigate activity in the subgenual anterior cingulate cortex (sgACC). Participants did not take any concomitant medication. Depression symptoms were assessed as a secondary outcome, at baseline, 24 hours and 7 days post-infusion.	KET (21)	i.v.	0.5 mg/kg
**Fu et al. 2020** (37)	224	138 (61,60%)	39,3 (12.91)	MADRS	The purpose of this double-blind, multicenter study was to evaluate the efficacy of intranasal esketamine (84 mg) compared with placebo in patients with MDD who were assessed to be at imminent risk for suicide. Patients received esketamine or placebo twice weekly for four weeks additionally to standard-of-care-treatment. Depression symptoms were assessed at baseline, 4h, 24h, 7d, 14d, 3w later.	ESK (112)	i.n.	84 mg
**Ionesco et al. 2021** (38)	227	136 (59.9%)	40.8 (13.07)	MADRS	This double-blind, randomized multicenter study examined the effect of esketamine in reduction of depression symptoms in patients with MDD with active suicide ideation. Participants received intranasal esketamine (84mg) or placebo twice weekly for four weeks additionally to standard-of-care treatment. Depression symptoms were assessed at baseline, 4h, 24h and 7d later.	ESK (114)	i.n.	84 mg
**Ionesco et al. 2019** (39)	26	10 (38.46%)	45.4 (12.4)	MADRS	This double-blind, randomized study included patients with severe major depressive disorder and current, chronic suicidal ideation. The participants received investigated intravenous ketamine (0.5 mg/kg) or placebo twice weekly over three weeks. Depression symptoms were assessed at baseline, 4-hours post-infusion and during a three-month follow-up phase.	KET (13)	i.v.	0.5 mg/kg
**Li et al. 2016^5^** (40)	32	24 (75.00%)	46.6 (10.56)	HDRS-17	This double-blind, randomized study examined the rapid effects of intravenous ketamine (0,2mg/kg or 0,5mg/kg) in comparison to a placebo on the prefrontal cortex and the amygdala activation in patients with treatment resistant depression. Depression symptoms were assessed at baseline, 40 minutes and 4h later.	KET (16)	i.v.	0.5 mg/kg
**Milak et al. 2020^6^** (41)	19	10 (52.63%)	42.6 (13.66)	MADRS HDRS-22	This double-blind, randomized study investigated the relationship of ketamine dose (0.1, 0.2, 0.3, 0.4, or 0.5 mg/kg) with magnetic resonance spectroscopy of Glx and GABA response in patients with MDD compared to placebo. Depression score was a secondary outcome in this study. Depression symptoms were assessed at baseline, 24h later.	KET (14)	i.v.	0.5 mg/kg 0.4 mg /kg
**Moayedi et al. 2023 (** 42 **)**	30	19 (63.33%)	28,5 (11.63)	BDI	This double-blind study compared the effect of intravenous ketamine (0,5mg/kg) and placebo in suicidal ideas and depression symptoms among patients with MDD. Depression symptoms were assessed at baseline, 2h, 24h and 3d later.	KET (15)	i.v.	0.5 mg/kg
**Singh et al. 2016a** (43)	30	18 (60.00%)	43 (11.5)	MADRS	This double-blind, randomized study investigated the efficacy and safety of a single treatment application with intravenous esketamine (0.2 or 0,4 mg/kg) in patients with treatment-resistant depression. Depression symptoms were assessed at baseline, 4h, 24h, 48h and 7d later.	ESK (20)	i.v.	0.2 mg/kg 0.4 mg/kg
**Singh et al. 2016b** (44)	67	(65.67%)	43.9 (11.0)	MADRS	This double-blind, randomized study evaluated the antidepressant effects of intravenous ketamine (0,5mg/kg) applied twice or thrice weekly for four weeks in comparison to placebo in patients with treatment-resistant depression (TRD). Depression symptoms were assessed at baseline, 3d, 7d, 14d later.	KET (35)	i.v.	0.5 mg/kg
**Takahashi et al. 2021** (45)	202	96 (47.52%)	43.4 (10.35)	MADRS	This double-blind, randomized study included patients with TRD and nonresponse to ≥ 1 but < 5 different antidepressants (ADs) in the current episode that also did not response to newly initiated oral AD for 6 weeks. These patients received intranasal esketamine (28; 56; or 84-mg) or placebo additionally to the initiated AD twice weekly for 4 weeks. Depression symptoms were assessed at baseline, 24h, 8d and14d later.	ESK (122)	i.n.	28 mg 56 mg 84 mg
**Tiger et al. 2020** (46)	30	12 (46,66%)	38.09 (n/a)	MADRS	This randomized placebo-controlled PET study examined the effect of single-dose ketamine intravenous infusion (0,5mg/kg) on serotonin receptor1B binding in patients with SSRI-resistant depression. Depression symptoms were assessed at baseline, directly after treatment.	KET (20)	i.v.	0.5 mg/kg
**Summary**	**1100**	**627** **(57.00%)**	**40.45** **(12.74)**	**-**	**-**	**-** **(593)**	**-**	**-**

^1^ESK, esketamine; KET, ketamine.

^2^i.n., intranasal; p.o., per oral; i.v., intravenous.

^3^Clinical doses were defined as ≥0,2 mg / kg esketamine due to the findings of the Singh et al. 2016a (43) study which showed statistically and clinically meaningful reduction of depression symptoms for both esketamine dose groups (.20 mg/kg and .40 mg/kg) compared to placebo. Respectively, clinical doses were defined as ≥0,4 mg/kg for ketamine.

^4^Participants receiving lanicemine (n=20) were excluded. In this study a total of 60 participants received either lanicemine (n=20), ketamine (n=21) or placebo (n=21).

^5^Participants receiving a subclinical dose of ketamine (0.2 mg / kg) were excluded from analysis. In this study a total of 48 patients received either ketamine in a subclinical dose of 0.2 mg / kg (n=16), a clinical dose of 0.5 mg / kg (n=16) or placebo (n=16).

^6^Participants receiving a subclinical dose of ketamine (0.1; 0.2 or 0.3 mg / kg) were excluded from analysis. In this study a total of 38 patients received either ketamine in a subclinical dose of 0.1 mg / kg (n=5), 0.2 mg / kg (n=6) ,0.3 mg / kg (n=5) or a clinical dose of 0.4 mg / kg (n=5) or 0.5 mg / kg (n=9) or placebo (n=5).

**Figure 3:**
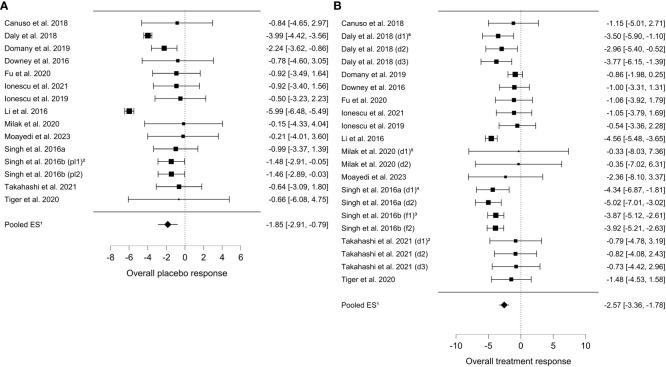
Overall placebo and treatment response. **(A)** Forest plot overall placebo response dpl = -1.85; z = -3.42; p < 0.001; [CI 95%, -2.9 to -0.79]; I² = 86.22%. (1) ES = effect size (cohen’s d). (2) Singh et al., 2016b (44): pl1 = first placebo group pl2 = second placebo group. **(B)** Forest plot overall treatment response dtr = -2.57; z =-6.36; p < 0.001; [CI 95% -3.36 to -1.78]; I² = 58.51%. (1) ES = effect size (cohen’s d). (2) Takahashi et al. (45),: d1 = 28 mg esketamine; d2 = 56 mg esketamine; d3 = 84mg esketamine. (3) Singh et al., 2016b (44): f1 = 2 x week; f2 = 3 x week. (4) Singh et al., 2016a (43): d1 = 0.2 mg/kg esketamine; d2 = 0.4 mg/kg esketamine. (5) Milak et al. (41),: d1 = 0.4 mg/kg ketamine; d2 = 0.5 mg/kg ketamine. (6) Daly et al., 2018 (34): d1 = 28 mg esketamine; d2 = 56 mg esketamine; d3 = 84mg esketamine.

The pooled effect size for the overall placebo response was: *d*pl = -1.85 (*z* = -3.42; *p* <.001; [CI 95%, -2.9 to -0.79]; *I*² = 86.22%) and for the overall treatment response: d_tr_ = -2.57 (z =-6.36; p < 0.001; [CI 95% -3.36 to -1.78]; I² = 58.51%) (**Figure 3**). These results indicate that 71,43% of the treatment response is replicated in the placebo group. This analysis included the comparison of different post-intervention time points spanning from 4 hours to 72 hours post-intervention. No significant interaction between the pooled effect size and the different time points was found in both treatment and placebo groups.

Subgroup analysis of 8 studies for the 7 days post-intervention time point resulted to the following pooled effect size for the placebo response: *d*_pl 7d_ = -1.98 (z = -3.02; *p* = .003; [CI 95%, -3.26 to -0.69], *I*² = 86.07%) and the treatment response: *d*_tr 7d_ = - 3.01 (*z* = -4.65; *p* <.001; [CI 95%, -4.28 to -1.74]; *I*² = 83,13%) was estimated. An overview is provided in the supplement (**Supplementary eFigure 1**). The placebo response accounts for 65,78% of the treatment effect. Subgroup analysis for the placebo response in studies with available data for the 40 minutes, 2 hours, 4 hours and 24 hours post-intervention time points respectively did not show any significant results. Subgroup analysis for studies with ketamine resulted to following placebo response d_pl ket_ = -1.92 (z = -2.47; p = .0014; [CI 95% -3.44 to -0.37]; I² = 83,94%) and treatment response: *d*_tr ket_ = -2.45 (z= -3.89; [CI 95% -3.69 to -1,21]; I² = 73,52%) (**Supplementary eFigure 2**). Subgroup analysis for studies with esketamine was also performed and resulted to following placebo response *d*_pl esk_ *=* -1.72 (z = -1.72; p = .023; [CI 95% -3.2 to -0,24]; I² = 67,88%) and treatment response: *d*_tr esk_ = -2.67 (z= -2,67; p <.001; [CI 95% - 3.70 to -1.649, I² = 33.80%) (**Supplementary eFigure 3**). According to this subgroup analysis the placebo response in studies with ketamine and in studies with esketamine accounts for 78% and 64% of the treatment response respectively.

Furthermore, we performed sensitivity analysis for the calculated pooled effect sizes by identifying outliers in the placebo groups and removing the respective studies from both groups before analysis. The estimated pooled effect size after sensitivity analysis for the overall placebo response was: *d* = -1.32 (*z* = -4.16; *p* <.001; [CI 95%, -1.94 to -0.70]; *I*² = 0%) and for the overall treatment response: *d* = -2.14 (z= -4.55; p <.001; [CI 95%, -3.07 to -1.22]; I² = 54.83%) (**Supplementary eFigure 4**). The overall placebo response accounts for 61.68% of the overall treatment response after sensitivity analysis. The estimated pooled effect size after sensitivity analysis for the placebo response 7 days post-intervention: *d* = -1.08 (*z* = -2.90; *p* = .004; [CI 95%, -1.80 to -0.35]; *I*² = 0%) and for the treatment response 7 days post-intervention: *d* = -2.48 (z = -3.08; *p* <.001; [CI 95% -4.06 to -0.90]; *I*² = 81.41%) (**Supplementary eFigure 5**). The placebo response accounts for 43.54% of the treatment response 7 days post-intervention.

**Figure 4:**
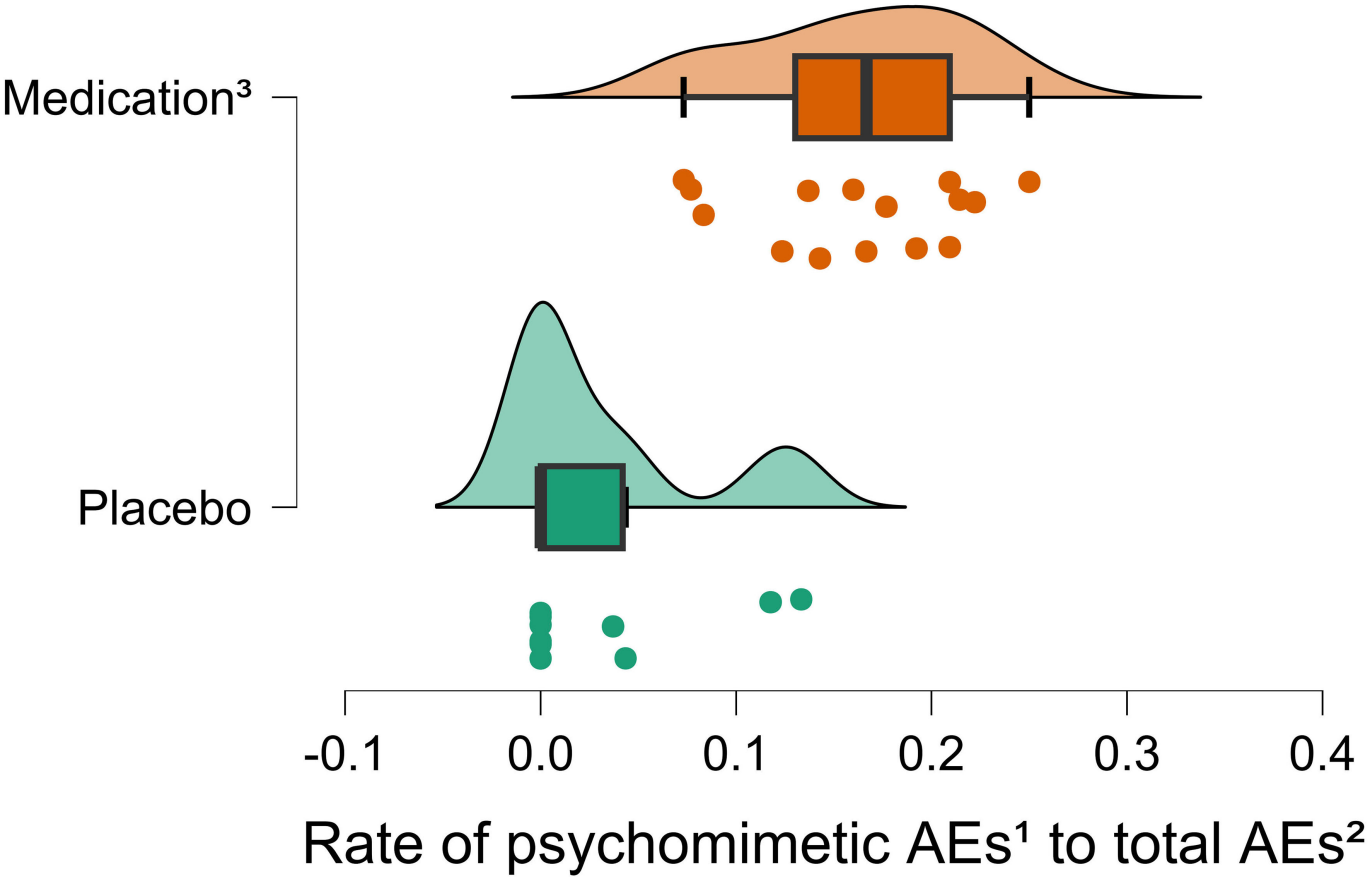
Rate of psychometic AEs to total AEs. Raincloud plot visualizing the results of the unpaired T-Test for the rate of psychomimetic AEs to total AEs between the medication and placebo groups. The rate of psychomimetic AEs to total AEs is significantly higher than in the placebo group t(21)= -5.95; p <.001. (1) Psychomimetic adverse events (AEs) include distinct medication-specific side effects like dissociation and hallucinations. (2) Total AEs include the summary of all reported AEs. (3) Medication = ketamine and esketamine.

Possible moderators of the placebo response such as sample size, age, sex, frequency of treatments, route of application, source of funding, country of study, year of publication, depressive symptoms at baseline, dosage and total number of the applications planned in the study, were investigated by performing meta-regression models. None of these above-mentioned variables were found to have a significant effect on the placebo response in the investigated studies. Adverse events were reported in 9 of the 14 included studies. A total of 1245 AEs (286 in the placebo and 959 in the medication group) were reported identified. A detailed overview of the reported AEs is provided in the supplement. We compared the rate of specific and psychomimetic AEs to the total AEs in the placebo and medication group. Medication specific AEs occur as expected significantly more often in the medication group *t*(14) = -2.67; *p*=.009 (**Supplementary eFigure 6**). Psychomimetic AEs are reported significantly more often in the medication group *t*(21) = -5.95; *p* <.001 (**Figure 4**).

## Discussion

This systematic review and meta-analysis included double-blind, randomized, placebo-controlled trials investigating the antidepressant effect of ketamine and esketamine in patients with MDD, with an overall placebo response of *d*_pl_ = -1.98. Our findings suggest that the placebo response accounts for 72% of the overall treatment response (*d*_tr_ = -2.57) reported in these studies.

Previous meta-analyses for placebo response in MDD only focused on oral antidepressants, our study however, is the first to address alternative routes of application and psychoactive antidepressants. Our findings are consistent with results from meta-analyses focused on oral antidepressants (1, 3). Moreover, our sensitivity analyses performed for the overall placebo and treatment response further highlight the robustness of our findings. Even though we opted for a conservative approach in our sensitivity analyses (avoiding overestimation of the placebo response by favoring the medication group in outlier removal) the placebo response it still accounts for 62% of the treatment response. The results of the placebo and the treatment response in studies with ketamine (**Figure 2**) and studies with esketamine (**Figure 3**) underline the results of our sensitivity analysis by showing that the placebo response accounts for 78% and 64% of the respective treatment response. These results also indicate a possible underestimation of the placebo response. The difference between ketamine and esketamine studies is possibly due to imbalance in residual heterogeneity in the esketamine studies treatment response (I²= 33%) and esketamine studies placebo response (I² = 68%) contrary to the balanced residual homogeneity in the placebo (84%) and treatment response (I² = 73%) of ketamine studies. We identified the small and differing sample sizes as a possible source of inhomogeneity.

In addition to sensitivity analysis, we also performed meta-regressions to investigate if the pooled effect sizes were influenced by differences in the study protocols (post-treatment time points, duration and number of treatments). Different intervention time points, frequency of treatments, route of application, dosage and total number of the applications planned did not interact significantly with the pooled effect size of the placebo and treatment response. In summary, our results indicate that 28-40% of the overall ketamine and esketamine treatment response can be attributed to factors other than the placebo response, like pharmacological effects. However, caution is advised in the interpretation of our findings in order to avoid an underestimation of psychopharmacological treatment effectivity since the drug–placebo differences in ketamine treatment for MDD are similar with the drug-placebo differences of general medicine drugs (47).

Seven days post-intervention the placebo response (*d*_pl 7d_ = -1.98) accounts for 66% of the treatment response (*d*_tr 7d_ = - 3.01), thus confirming the before mentioned results. After sensitivity analysis, the placebo response accounted for 43% of the treatment response. However, these results should be interpreted with caution due to the high imbalance in heterogeneity in the treatment (*I*² = 83%) and the placebo group (*I*² = 0%). A possible explanation for the high residual heterogeneity in the treatment group lies in the varying number of applied interventions per study spanning from 1 to 3 applications per week.

Our risk of bias calculation for the included studies indicates an overall high risk of bias in favor of the medication groups. These results suggest a possible underestimation of the placebo response. We identified insufficient blinding due to the distinct psychomimetic effects of ketamine and esketamine as the main source of possible assessor and participant bias. Some study designs reduced assessor bias by assigning different assessors for safety and efficacy rating. However, there is a high probability that the participants receiving medication were unblinded. This is also indicated by our analysis of the reported AEs; psychomimetic AEs show a significantly higher rate in the medication group *t*(21) = -5.95; *p* <.001 (**Figure 4**) than in the placebo group. Moreover, insufficient blinding has been addressed as a limitation in a number of double-blind trials investigating the effects of ketamine (48) and esketamine (49, 50) in patients with MDD.

The high placebo response in MDD treatment with NMDA receptor antagonists shown in our study is clinically relevant and should be considered in clinical practice to enhance patient treatment outcome. Additionally, from a clinical perspective it is important to take possible side-effects like psychomimetic AEs or other AEs like sedation or somnolence into consideration when discussing treatment options with patients. This is particularly relevant for patient groups, like elderly patients, whose tolerance for such symptoms could be compromised.

The growing interest of the scientific community regarding psychedelic research (51), the increasing concerns about data quality of the studies (52), as well as the recent first worldwide authorization of psychedelic substances for the treatment of mental diseases from the Australian Therapeutic Goods Administration (TGA) (53) show the high relevance of our findings and highlight the need for further optimization of study designs as also described in the previous literature (54). Controlling for expectation with a 2x2 factorial design which systematically crosses the intervention (psychoactive drug, placebo) with the standardized induced expectation (high or low expectation to receive medication or placebo) (55) would enhance the quality of the results. To date we have identified only one and still ongoing study investigating the effects of esketamine in patients suffering from MDD with the above-mentioned study design (56) and no study with ketamine. Furthermore, data quality would benefit from interventions designed to neutralize subject expectations (57). For instance, a double-blind controlled study by Cohen et al. (58) including patients with MDD and schizophrenia showed that providing study participants information about factors contributing to the placebo response before each measurement of the primary outcome can significantly reduce the placebo response. Moreover, other suggestions include routinely measuring de-blinding and expectancy (59); implementation of independent raters for efficacy and safety assessments (60) and use of active placebos (61).

Limitations of this study include the high heterogeneity of the included studies in some outcomes. Causes of heterogeneity are (1) the differences in study protocols of the included studies, (2) the synthesis of similar but yet differing interventions (ketamine and esketamine), (3) the overall high risk of bias of the included studies and (4) that studies allowed concomitant antidepressant medication. The first cause could be mitigated by a future larger meta-analysis, the second by performing separate meta-analyses for each intervention and the third by optimizing future study designs of double-blind RCTs investigating the treatment response of psychoactive substances. Lastly, the forth cause can be alleviated by including naturalistic study arms. We excluded studies that experimentally evaluated the combined intervention of a particular antidepressant with ketamine or esketamine but included studies allowing parallel standard of care. An advantage of this approach is that it provides a more representative patient population sample by facilitating the inclusion of patients with treatment resistant depression (TRD) while minimizing noise from the concomitant medication. Moreover, this approach prevented an inflation of placebo response because although patients with TRD are associated with high placebo response rates (62) these are still lower in comparison to patients with non-TRD (63). Despite the reported study limitations, the robustness of our findings is supported by similar findings in the literature and by the results of the sensitivity and subgroup analysis.

This meta-analysis concludes that the placebo response accounts for 62-71% of the treatment response in the included double-blind RCTs examining the antidepressant effects of ketamine and esketamine in patients with MDD. Furthermore, insufficient blinding in the included studies pose an important source of bias. Optimization of future study designs in trials with psychoactive substances is urgently needed. The placebo response plays a significant role in the treatment of depression and should be used for the benefit of the patients in clinical practice.

## Data availability statement

The original contributions presented in the study are included in the article/**Supplementary Material**. Further inquiries can be directed to the corresponding author.

## Author contributions

AM: Conceptualization, Data curation, Formal analysis, Investigation, Methodology, Project administration, Validation, Visualization, Writing – original draft, Writing – review & editing. MW: Conceptualization, Methodology, Writing – review & editing. LN: Data curation, Formal analysis, Investigation, Writing – review & editing. CY: Writing – review & editing. WR: Writing – review & editing. SH: Writing – review & editing. IF: Funding acquisition, Writing – review & editing. TK: Funding acquisition, Methodology, Project administration, Resources, Supervision, Writing – review & editing.
