# Supplementary material for: Hype or hope? High placebo response in major depression treatment with ketamine and esketamine: a systematic review and meta-analysis
Source: Front Psychiatry. 2024 Mar 8;15:1346697. doi: 10.3389/fpsyt.2024.1346697 (PMC10957753; doi:10.3389/fpsyt.2024.1346697)
Supplement: Supplementary file 1 [file DataSheet_1.docx]

# Supplementary material

**eTable 1: Search strategy**

**eTable 2: Summary of AEs in the placebo group**

**eTable 3: Summary of AEs in the medication group**

**eTable 4: Non-Psychomimetic AEs in the placebo group**

**eTable 5: Psychomimetic AEs in the placebo group**

**eTable 6: Non-Psychomimetic AEs in the medication group**

**eTable 7: Psychomimetic AEs in the medication group**

**eFigure 1: Placebo and treatment response 7 days post-intervention**

**eFigure 2: Placebo and treatment response in ketamine studies**

**eFigure 3: Placebo and treatment response in esketamine studies**

**eFigure 4: Overall placebo and treatment response after sensitivity analysis**

**eFigure 5: Placebo and treatment response 7 days post-intervention after sensitivity analysis**

**eFigure 6: Rate of specific AEs to the total AEs**

# A. Search strategy

We searched following databases: Embase, PubMed/MEDLINE, the Cochrane Central Register of Controlled Trials (CENTRAL) and PsycINFO.

Following search terms were used: (placebo) AND (esketamin*) OR (ketamin*) AND (depress*). The search was conducted on August 3th 2022. A total of 5017 record were identified. A complementary manual search using Google Scholar was performed on March 17^th^ 2023.

## eTable 1 Search strategy

| **Database** | **Search String** |
| --- | --- |
| Embase | (depress*:ti,ab,kw AND esketamin*:ti,ab,kw OR ketamin*:ti,ab,kw) AND placebo:ti,ab,kw |
| PubMed/MEDLINE | (((depress*[Title/Abstract]) AND (ketamin*[Title/Abstract])) OR (esketamin*[Title/Abstract])) AND (placebo[Title/Abstract]) |
| CENTRAL | depress* AND ketamin* OR esketamin* AND placebo |
| PsycINFO* | AB depress* AND AB ketamin* OR AB esktetamin* AND AB placebo |

*The search was conducted through the Ebsco-Plattform

# B. Adverse events

Adverse events (AEs) were extracted from the published articles or the AE section of the study protocols and were categorized in specific and non-specific AEs. Specific AEs included medication specific side effects as reported in the information leaflet for ketamine (1) and esketamine (2). The remaining AEs were categorized as unspecific.

Furthermore, specific AEs were divided in two clusters of AEs: psychomimetic and non psychomimetic AEs. Psychomimetic AEs included symptoms distinctly attributed to ketamine and esketamine like dissociation and other psychiatric symptoms. The rest of the specific AEs were categorized as non-psychomimetic AEs.

An overview of the AE synthesis and variable description for specific AEs is provided at **eTable 2**. The summary of the AEs included in the placebo and medication group are shown in **eTable 3** and **eTable 4** respectively.A detailed report of the included non-psychomimetic and psychomimetic AEs is provided in eTable 5 and eTable 6 for the placebo groups and eTable 7 and eTable 8 for the medication groups.

The performed AE-synthesis was not included in the preregistration protocol. The idea to quantify study blinding developed after completion of the protocol.

## eTable 2: Summary of AEs in the placebo group

| **Summary of AEs in the placebo group** | | | | |
| --- | --- | --- | --- | --- |
| **Study** | **N (participants)** | **Summary specific AEs** | **Summary unspecific AEs** | **Summary**  **total AEs** |
| Canuso et al. 2018 | 31 | 12 | 18 | 30 |
| Daly et al. 2018 | 33 | 5 | 18 | 23 |
| Downey et al. 2016 | 19 | 1 | 1 | 2 |
| Domany et al. 2019 | 18 | 4 | 2 | 6 |
| Fu et al. 2020 | 112 | 46 | 62 | 108 |
| Ionescu et al. 2021 | 113 | 0 | 9 | 9 |
| Singh et al. 2016a | 10 | 2 | 3 | 5 |
| Singh et al. 2016b (pl1)^1^ | 16 | 2 | 17 | 19 |
| Singh et al. 2016b (pl2) | 16 | 2 | 14 | 16 |
| Takahashi et al. 2021 | 80 | 28 | 40 | 68 |
| **Summary** | **448** | **102** | **184** | **286** |

**^1^** Singh et al. 2016b: pl1 = first placebo group; pl2 = second placebo group

## eTable 3: Summary of AEs in the medication group

| **Summary of AEs in the medication group** | | | | |
| --- | --- | --- | --- | --- |
| **Study** | **N**  **(participants)** | **Summary specific AEs** | **Summary unspecific AEs** | **Summary**  **total AEs** |
| Canuso et al. 2018 | 35 | 59 | 30 | 89 |
| Daly et al. 2018 (d1)^4^ | 11 | 13 | 12 | 25 |
| Daly et al. 2018 (d2) | 11 | 24 | 19 | 43 |
| Daly et al. 2018 (d3) | 12 | 25 | 18 | 43 |
| Downey et al. 2016 | 21 | 3 | 1 | 4 |
| Domany et al. 2019 | 22 | 7 | 6 | 13 |
| Fu et al. 2020 | 112 | 136 | 105 | 241 |
| Ionescu et al. 2021 | 114 | 1 | 5 | 6 |
| Singh et al. 2016a (d1)^3^ | 9 | 6 | 6 | 12 |
| Singh et al. 2016a (d2) | 11 | 14 | 12 | 26 |
| Singh et al. 2016b (f1)^2^ | 18 | 24 | 27 | 51 |
| Singh et al. 2016b (f2) | 17 | 15 | 23 | 38 |
| Takahashi et al. 2021 (d1)^1^ | 40 | 53 | 28 | 81 |
| Takahashi et al. 2021 (d2) | 41 | 72 | 68 | 140 |
| Takahashi et al. 2021 (d3) | 40 | 73 | 74 | 147 |
| **Summary** | **514** | **525** | **434** | **959** |

**^1^** Takahashi et al. 2021: d1 = 28 mg esketamine; d2 = 56 mg esketamine; d3 = 84mg esketamine

**^2^** Singh et al. 2016b: f1 = 2 x week; f2 = 3 x week

**^3^** Singh et al. 2016a: d1 = 0,2 mg/kg esketamine d2 = 0,4 mg/kg esketamine

**^4^** Daly et al. 2018: d1 = 28 mg esketamine; d2 = 56 mg esketamine; d3 = 84mg esketamine

## eTable 4: Non-Psychomimetic AEs in the placebo group

| **Non-Psychomimetic AEs in the placebo group** | | | | | | | | | | | | | |
| --- | --- | --- | --- | --- | --- | --- | --- | --- | --- | --- | --- | --- | --- |
| ***Study*** | **Gastrointestinal symptoms** | | **CNS**  **symptoms** | | | | **distorted**  **sight** | | | **sensory**  **symptoms** | | | |
|  | *nausea* | *vomiting* | *vertigo* | *dizziness* | *sedation* | *somnolence* | *blurred vision* | *altered vision depth* | *diplopia* | *paresthesia* | *paresthesia oral* | *hypoesthesia* | *hypoesthesia oral* |
| Canuso et al. 2018 | 1 | 0 | 0 | 4 | 2 | n/a | 0 | n/a | n/a | 1 | n/a | n/a | n/a |
| Daly et al. 2018 | 3 | n/a | 0 | 1 | 0 | n/a | 0 | n/a | n/a | n/a | n/a | n/a | n/a |
| Downey et al. 2016 | n/a | n/a | n/a | n/a | n/a | 1 | n/a | n/a | n/a | n/a | n/a | n/a | n/a |
| Domany et al. 2019 | 2 | 1 | n/a | 1 | n/a | n/a | 0 | n/a | n/a | n/a | n/a | n/a | n/a |
| Fu et al. 2020 | 15 | 7 | 1 | 10 | 2 | n/a | 5 | n/a | n/a | n/a | n/a | 2 | n/a |
| Ionescu et al. 2021 | n/a | n/a | n/a | n/a | n/a | n/a | n/a | n/a | n/a | n/a | n/a | n/a | n/a |
| Singh et al. 2016a | 2 | 0 | 0 | 0 | n/a | n/a | n/a | n/a | n/a | 0 | n/a | n/a | n/a |
| Singh et al. 2016b (p1)^1^ | 1 | n/a | n/a | 1 | n/a | n/a | 0 | 0 | n/a | 0 | 0 | 0 | 0 |
| Singh et al. 2016 (pl2) | 2 | n/a | n/a | 0 | n/a | n/a | 0 | 0 | n/a | 0 | 0 | 0 | 0 |
| Takahashi et al. 2021 | 7 | 3 | 1 | 5 | 0 | n/a | n/a | n/a | 0 | n/a | n/a | 4 | 0 |
| **Summary** | **44** | | **29** | | | | **5** | | | **7** | | | |

**^1^** Singh et al. 2016b: pl1 = first placebo group; pl2 = second placebo group

## eTable 5: Psychomimetic AEs in the placebo group

| **Psychomimetic AEs in the placebo group** | | | | | | | | | | | | | | |
| --- | --- | --- | --- | --- | --- | --- | --- | --- | --- | --- | --- | --- | --- | --- |
| **Study** | **Psychiatric**  **symptoms** | | | | | | | **Feeling abnormal** | **Dissociation** | | | | | |
|  | visual  hallucinations | tactile hallucinations | nightmares | paranoia | restlessness | feeling drunk | mental impairment |  | dissociation | dissociation symptoms | dissociation disorder | dissociative disorder | derealization disorder |  |
| Canuso et al. 2018 | n/a | n/a | n/a | n/a | n/a | n/a | n/a | n/a | 4 | n/a | n/a | n/a | n/a |  |
| Daly et al. 2018 | n/a | n/a | n/a | n/a | n/a | n/a | n/a | 0 | 1 | n/a | n/a | 0 | n/a |  |
| Downey et al. 2016 | n/a | n/a | n/a | n/a | n/a | 0 | n/a | n/a | n/a | n/a | n/a | n/a | n/a |  |
| Domany et al. 2019 | n/a | n/a | n/a | n/a | n/a | n/a | n/a | n/a | 0 | n/a | n/a | n/a | n/a |  |
| Fu et al. 2020 | n/a | n/a | n/a | n/a | n/a | n/a | n/a | n/a | 4 | n/a | n/a | n/a | n/a |  |
| Ionescu et al. 2021 | n/a | n/a | n/a | n/a | n/a | n/a | n/a | n/a | n/a | n/a | n/a | n/a | 0 |  |
| Singh et al. 2016a | n/a | n/a | n/a | n/a | n/a | n/a | n/a | n/a | 0 | n/a | n/a | n/a | n/a |  |
| Singh et al. 2016b (pl1)^1^ | 0 | 0 | 0 | 0 | 0 | n/a | n/a | 0 | 0 | n/a | 0 | n/a | n/a |  |
| Singh et al. 2016 (pl2) | 0 | 0 | 0 | 0 | 0 | n/a | n/a | 0 | 0 | n/a | 0 | n/a | n/a |  |
| Takahashi et al. 2021 | 0 | n/a | n/a | n/a | n/a | 1 | 0 | n/a | 7 | n/a | n/a | n/a | 0 |  |
| **Summary** | **1** | | | | | | | **0** | **16** | | | | | |

**^1^** Singh et al. 2016b: pl1 = first placebo group; pl2 = second placebo group

| **Non-Psychomimetic AEs in the medication group** | | | | | | | | | | | | | | |
| --- | --- | --- | --- | --- | --- | --- | --- | --- | --- | --- | --- | --- | --- | --- |
| **Study** | **Gastrointestinal symptoms** | | **CNS**  **symptoms** | | | | **distorted**  **sight** | | | **sensory**  **symptoms** | | | | |
|  | nausea | vomiting | vertigo | dizziness | sedation | somnolence | blurred vision | altered vision depth | diplopia | paresthesia | paresthesia oral | hypoesthesia | hypoesthesia oral |  |
| Canuso et al. 2018 | 13 | 7 | 4 | 12 | 6 | n/a | n/a | n/a | n/a | 6 | n/a | n/a | n/a |  |
| Daly et al. 2018 (d1)^4^ | 2 | n/a | 2 | 4 | 1 | n/a | 0 | n/a | n/a | n/a | n/a | n/a | n/a |  |
| Daly et al. 2018 (d2) | 4 | n/a | 1 | 8 | 2 | n/a | 0 | n/a | n/a | n/a | n/a | n/a | n/a |  |
| Daly et al. 2018 (d3) | 4 | n/a | 1 | 8 | 1 | n/a | 2 | n/a | n/a | n/a | n/a | n/a | n/a |  |
| Downey et al. 2016 | n/a | n/a | n/a | n/a | n/a | 1 | n/a | n/a | n/a | n/a | n/a | 1 | n/a |  |
| Domany et al. 2019 | 1 | 0 | n/a | 4 | n/a | n/a | 1 | n/a | n/a | n/a | n/a | n/a | n/a |  |
| Fu et al. 2020 | 23 | 8 | 7 | 40 | 7 | n/a | 10 | n/a | n/a | n/a | n/a | 8 | n/a |  |
| Ionescu et al. 2021 | n/a | n/a | n/a | n/a | n/a | n/a | n/a | n/a | n/a | n/a | n/a | n/a | n/a |  |
| Singh et al. 2016a (d1)^3^ | 3 | 1 | 0 | 1 | n/a | n/a | n/a | n/a | n/a | 0 | n/a | n/a | n/a |  |
| Singh et al. 2016a (d2) | 3 | 1 | 2 | 1 | n/a | n/a | n/a | n/a | n/a | 2 | n/a | n/a | n/a |  |
| Singh et al. 2016b (f1)^2^ | 3 | n/a | n/a | 4 | n/a | n/a | 2 | 0 | n/a | 1 | 1 | 0 | 1 |  |
| Singh et al. 2016b (f2) | 4 | n/a | n/a | 3 | n/a | n/a | 0 | 1 | n/a | 2 | 0 | 1 | 1 |  |
| Takahashi et al. 2021 (d1)^1^ | 7 | 1 | 4 | 11 | 1 | n/a | n/a | n/a | 1 | n/a | n/a | 7 | 3 |  |
| Takahashi et al. 2021 (d2) | 7 | 3 | 7 | 18 | 4 | n/a | n/a | n/a | 3 | n/a | n/a | 8 | 2 |  |
| Takahashi et al. 2021 (d3) | 8 | 4 | 8 | 15 | 5 | n/a | n/a | n/a | 0 | n/a | n/a | 5 | 2 |  |
| **Summary** | **107** | | **193** | | | | **20** | | | **51** | | | | |

## eTable 6: Non-Psychomimetic AEs in the medication group

**^1^** Takahashi et al. 2021: d1 = 28 mg esketamine; d2 = 56 mg esketamine; d3 = 84mg esketamine

**^2^** Singh et al. 2016b: f1 = 2 x week; f2 = 3 x week

**^3^** Singh et al. 2016a: d1 = 0,2 mg/kg esketamine d2 = 0,4 mg/kg esketamine

**^4^** Daly et al. 2018: d1 = 28 mg esketamine; d2 = 56 mg esketamine; d3 = 84mg esketamine

| **Psychomimetic AEs in the medication group** | | | | | | | | | | | | | |
| --- | --- | --- | --- | --- | --- | --- | --- | --- | --- | --- | --- | --- | --- |
| **Study** | **psychiatric disorders** | | | | | | | **feeling abnormal** | **dissociation** | | | | |
|  | visual  hallucinations | tactile hallucinations | nightmares | paranoia | restlessness | feeling drunk | mental impairment |  | dissociation | dissociation symptoms | dissociation disorder | dissociative disorder | derealization disorder |
| Canuso et al. 2018 | n/a | n/a | n/a | n/a | n/a | n/a | n/a | n/a | 11 | n/a | n/a | n/a | n/a |
| Daly et al. 2018 (d1)^4^ | n/a | n/a | n/a | n/a | n/a | n/a | n/a | 2 | 0 | n/a | n/a | 2 | n/a |
| Daly et al. 2018 (d2) | n/a | n/a | n/a | n/a | n/a | n/a | n/a | 1 | 7 | n/a | n/a | 1 | n/a |
| Daly et al. 2018 (d3) | n/a | n/a | n/a | n/a | n/a | n/a | n/a | 1 | 4 | n/a | n/a | 4 | n/a |
| Downey et al. 2016 | n/a | n/a | n/a | n/a | n/a | 1 | n/a | n/a | n/a | n/a | n/a | n/a | n/a |
| Domany et al. 2019 | n/a | n/a | n/a | n/a | n/a | n/a | n/a | n/a | 1 | n/a | n/a | n/a | n/a |
| Fu et al. 2020 | n/a | n/a | n/a | n/a | n/a | n/a | n/a | n/a | 33 | n/a | n/a | n/a | n/a |
| Ionescu et al. 2021 | n/a | n/a | n/a | n/a | n/a | n/a | n/a | n/a | n/a | n/a | n/a | n/a | 1 |
| Singh et al. 2016a (d1)^3^ | n/a | n/a | n/a | n/a | n/a | n/a | n/a | n/a | 1 | n/a | n/a | n/a | n/a |
| Singh et al. 2016a (d2) | n/a | n/a | n/a | n/a | n/a | n/a | n/a | n/a | 5 | n/a | n/a | n/a | n/a |
| Singh et al. 2016b (f1)^2^ | 1 | 1 | 1 | 1 | 1 | n/a | n/a | 1 | 5 | n/a | 1 | n/a | n/a |
| Singh et al. 2016b (f2) | 1 | 0 | 0 | 0 | 0 | n/a | n/a | 1 | 1 | n/a | 0 | n/a | n/a |
| Takahashi et al. 2021 (d1)^1^ | 0 | n/a | n/a | n/a | n/a | 1 | 3 | n/a | 14 | n/a | n/a | n/a | 0 |
| Takahashi et al. 2021 (d2) | 1 | n/a | n/a | n/a | n/a | 5 | 2 | n/a | 10 | n/a | n/a | n/a | 2 |
| Takahashi et al. 2021 (d3) | 2 | n/a | n/a | n/a | n/a | 2 | 0 | n/a | 22 | n/a | n/a | n/a | 0 |
| **Summary** | **23** | | | | | | | **6** | **125** | | | | |

## eTable 7: Psychomimetic AEs in the medication group

**^1^** Takahashi et al. 2021: d1 = 28 mg esketamine; d2 = 56 mg esketamine; d3 = 84mg esketamine

**^2^** Singh et al. 2016b: f1 = 2 x week; f2 = 3 x week

**^3^** Singh et al. 2016a: d1 = 0,2 mg/kg esketamine d2 = 0,4 mg/kg esketamine

**^4^** Daly et al. 2018: d1 = 28 mg esketamine; d2 = 56 mg esketamine; d3 = 84mg esketamin

## **eFigure 1: Placebo and treatment response 7 days post-intervention**

**eFigure 1 Part A: Forest plot placebo response (7 days post-intervention) dpl7d = -1.98; z = -3.02; p = .003; [CI 95%, -3.26 to -0.69], I² = 86.07 %**


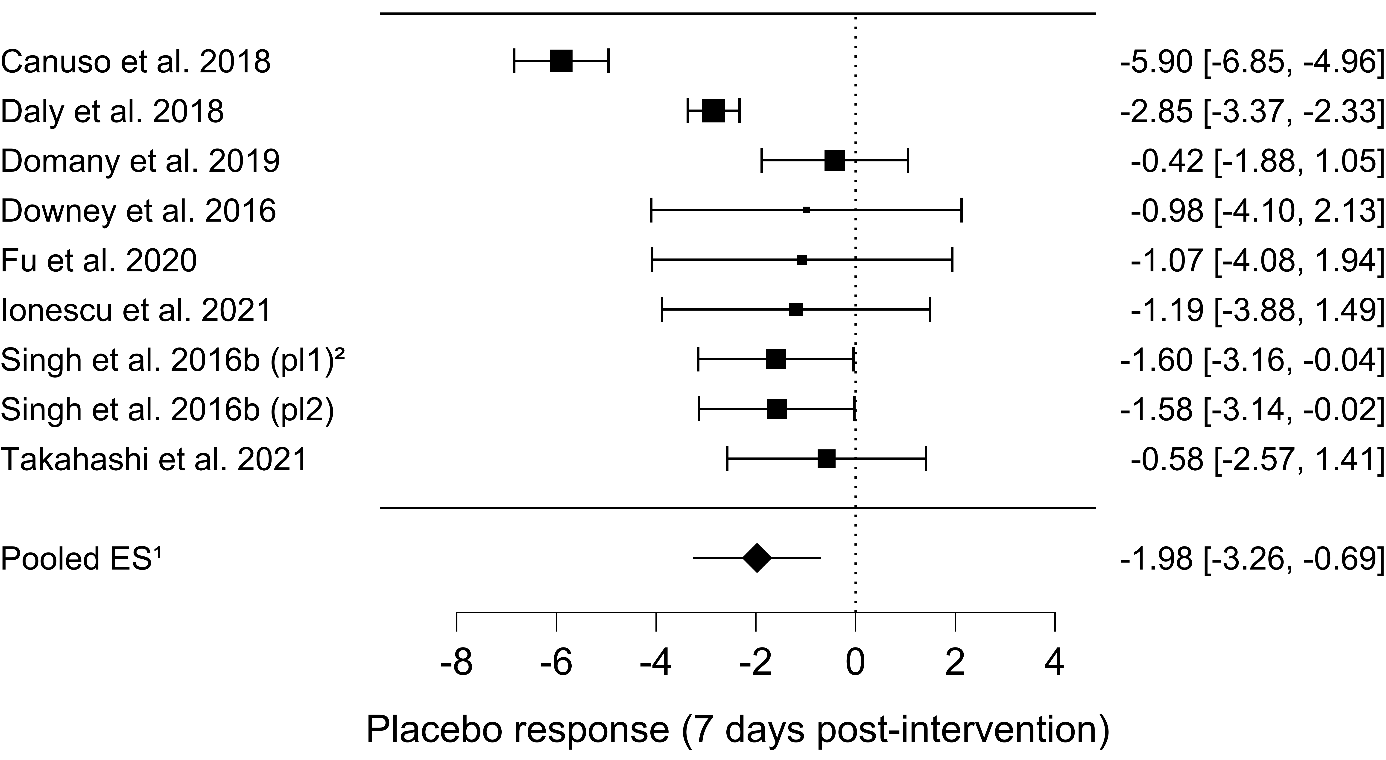


(1) ES = effect size (cohen‘s d)

(2) Singh et al. 2016b: pl1 = first placebo group pl2 = second placebo group

**eFigure 1 Part B: Forest plot treatment response (7 days post-intervention) dtr7d = - 3.01; z = -4.65; p < .001; [CI 95%, -4.28 to -1.74]; I² = 83,13%**


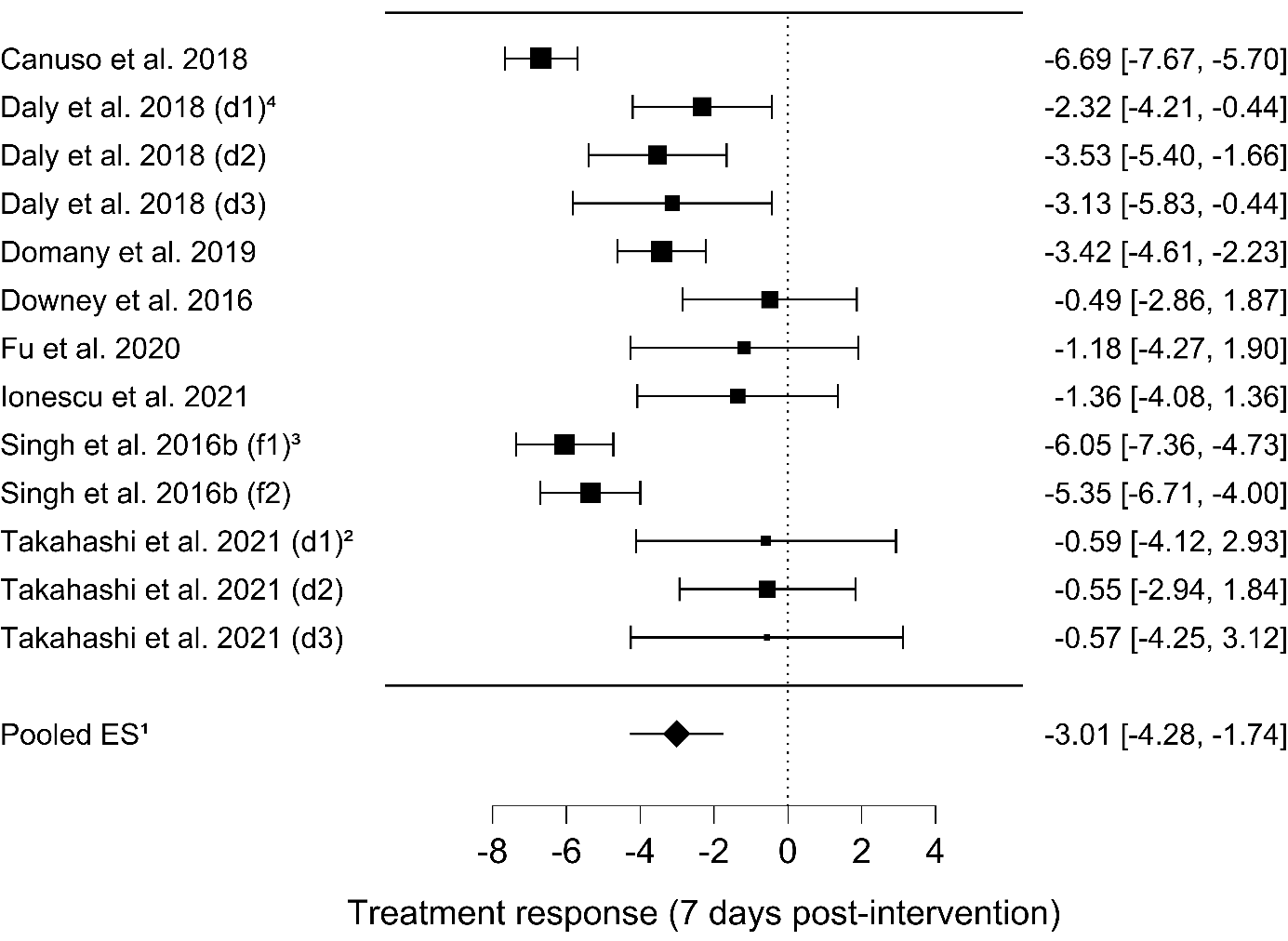


(1) ES = effect size (cohen‘s d)

(2) Takahashi et al. 2021: d1 = 28 mg esketamine ; d2 = 56 mg esketamine ; d3 = 84mg esketamine

(3) Singh et al. 2016b: f1 =  2 x week; f2 = 3 x week

(4) Daly et al. 2018: d1 = 28 mg esketamine ; d2 = 56 mg esketamine ; d3 = 84mg esketamine

## **eFigure 2: Placebo and treatment response in ketamine studies**

**eFigure 2 Part A: Forest plot placebo response in ketamine studies dpl ket = - 1.92; z = -2.47; p = .0014; [CI 95% -3.44 to -0.37]; I² = 83,94 %**


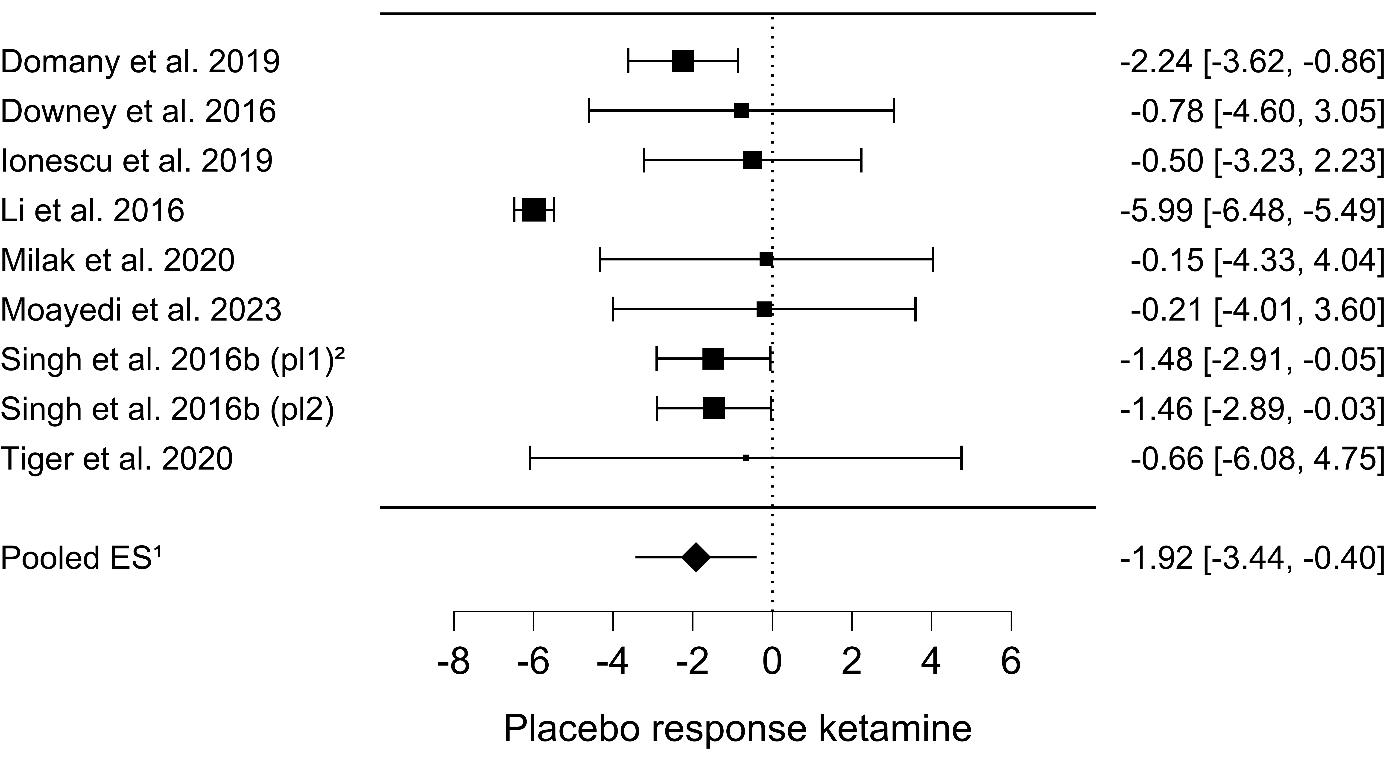


(1) ES = effect size (cohen‘s d)

(2) Singh et al. 2016b: pl1 = first placebo group pl2 = second placebo

**eFigure 2 Part B: Forest plot treatment response in ketamine studies dtr ket = -2.45; z= -3.89; [CI 95% -3.69 to -1,21]; I² = 73,52 %**


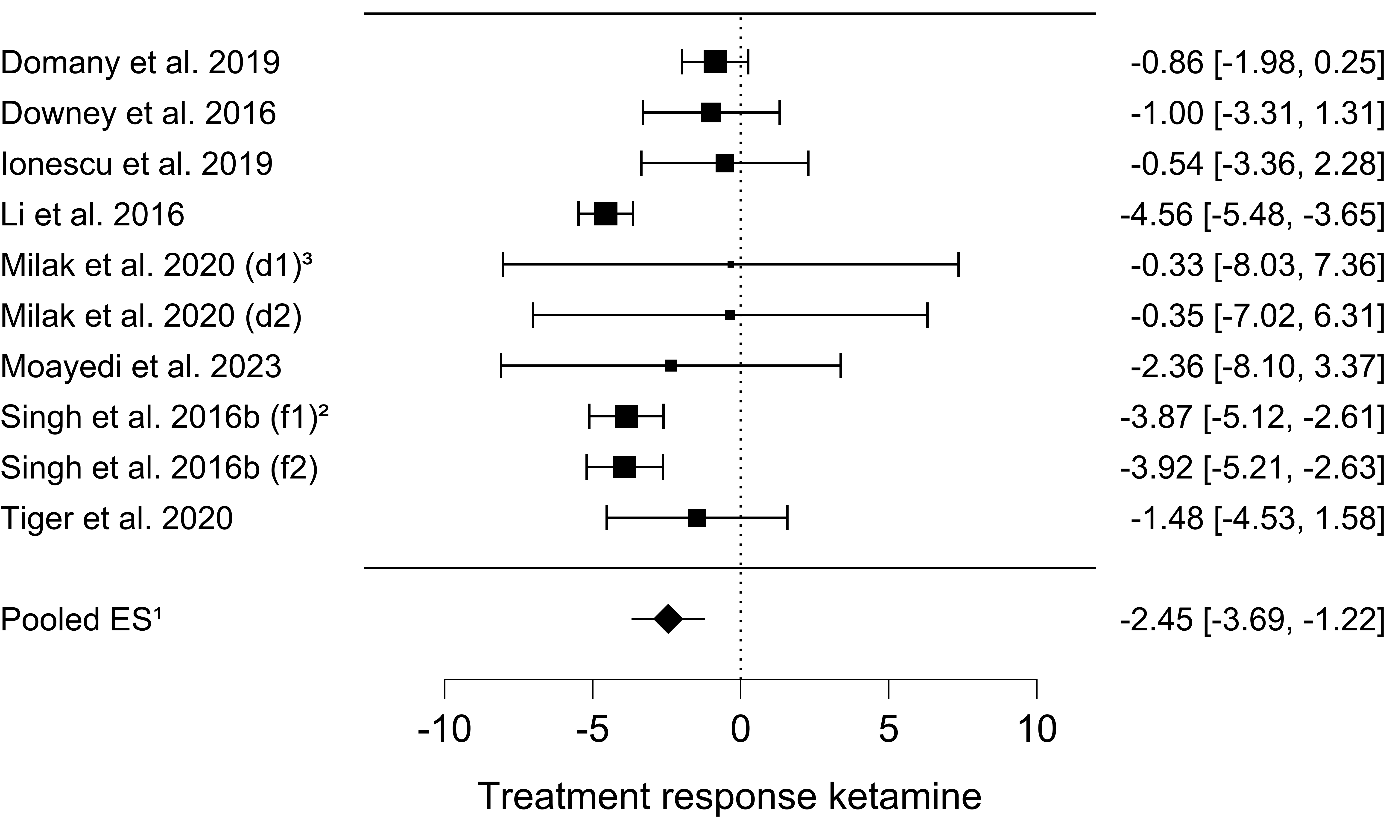
 (1) ES = effect size (cohen‘s d) (2) Singh et al. 2016b: f1 =  2 x week; f2 = 3 x week (3) Milak et al 2020: d1 = 0.4 mg/kg ketamine; d2 = 0.5 mg/kg ketamine

## **eFigure 3: Placebo and treatment response in esketamine studies**

**eFigure 3 Part A: Forest plot placebo response in esketamine studies d_pl esk_ = *=* -1.72; z = -1.72; p = .023; [CI 95% -3.2 to -0.24]; I² = 67,88 %**


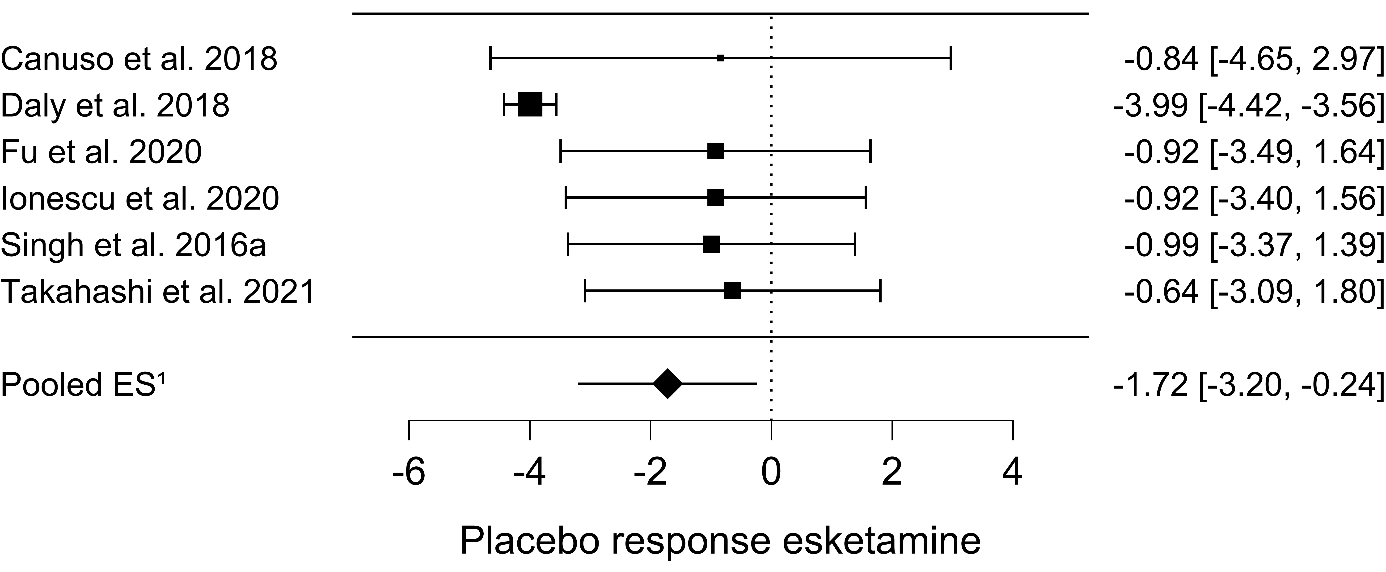

(1) ES = effect size (cohen‘s d)

**eFigure 3 Part A: Forest plot treatment response in esketamine studies d_tr esk_= -2.67; z= -2,67; p < .001; [CI 95% - 3.71 to -1.64], I² = 33.80 %**


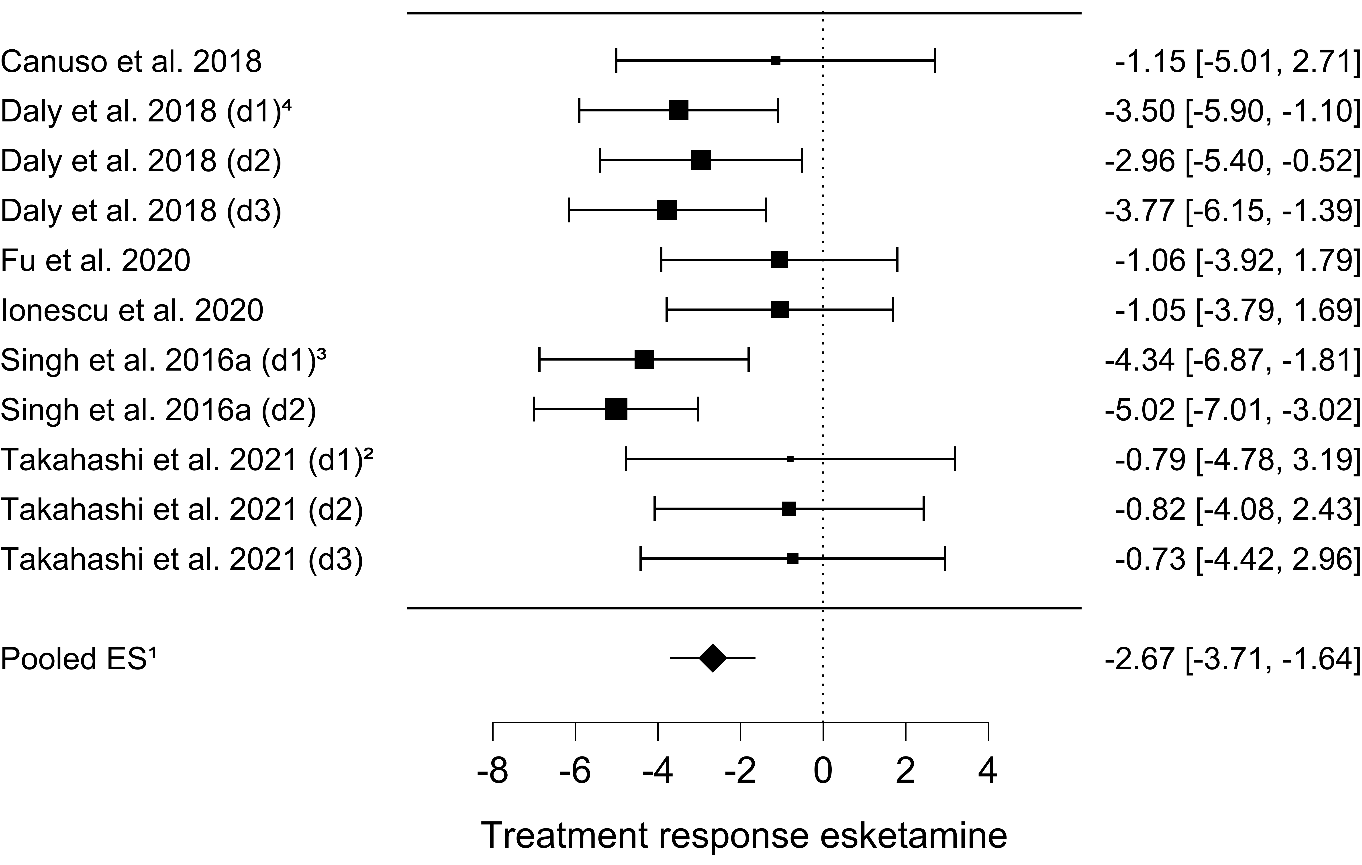


(1) ES = effect size (cohen‘s d)

(2) Takahashi et al. 2021: d1 = 28 mg esketamine ; d2 = 56 mg esketamine ; d3 = 84mg esketamine

(3) Singh et al. 2016a: d1 =  0,2 mg / kg ; d2 = 0,4 mg / kg

(4) Daly et al. 2018: d1 = 28 mg esketamine ; d2 = 56 mg esketamine ; d3 = 84mg esketamine

## eFigure 4: Overall placebo and treatment response after sensitivity analysis

**eFigure 4 Part A: Forest plot after sensitivity analysis of overall placebo response dpl(sens) = -1.32; z = -4.16; p < .001; [CI 95%, -1.94 to -0.70]; I² = 0 %**


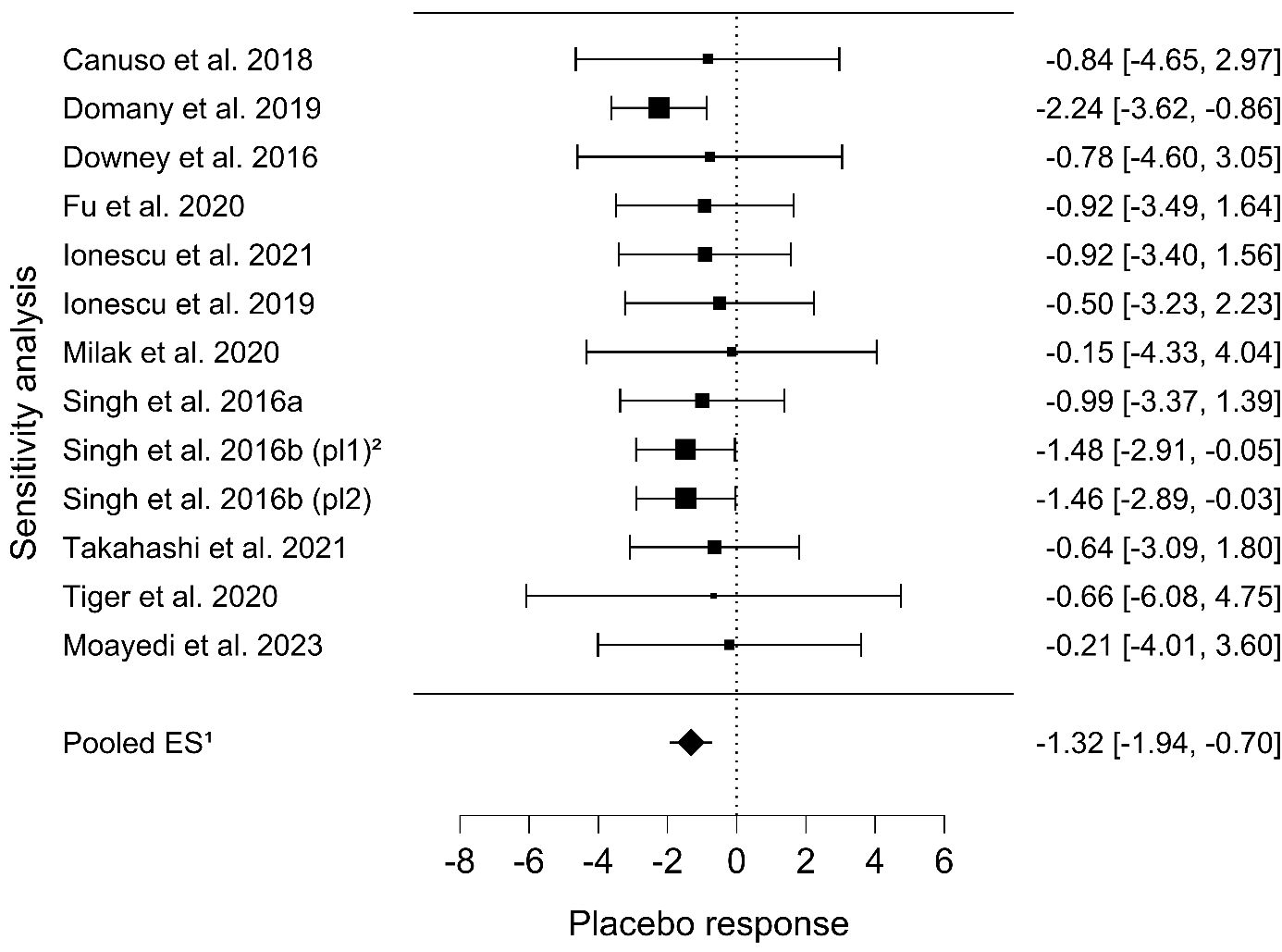


(1) ES = effect size (cohen‘s d)

(2) Singh et al. 2016b: pl1 = first placebo group pl2 = second placebo group

**eFigure ~~4~~. Part B: Forest plot after sensitivity analysis of overall treatment response dtr(sens) = -2.14; z= -4.55; p < .001; [CI 95%, -3.07 to -1.22]; I² = 54.83 %**


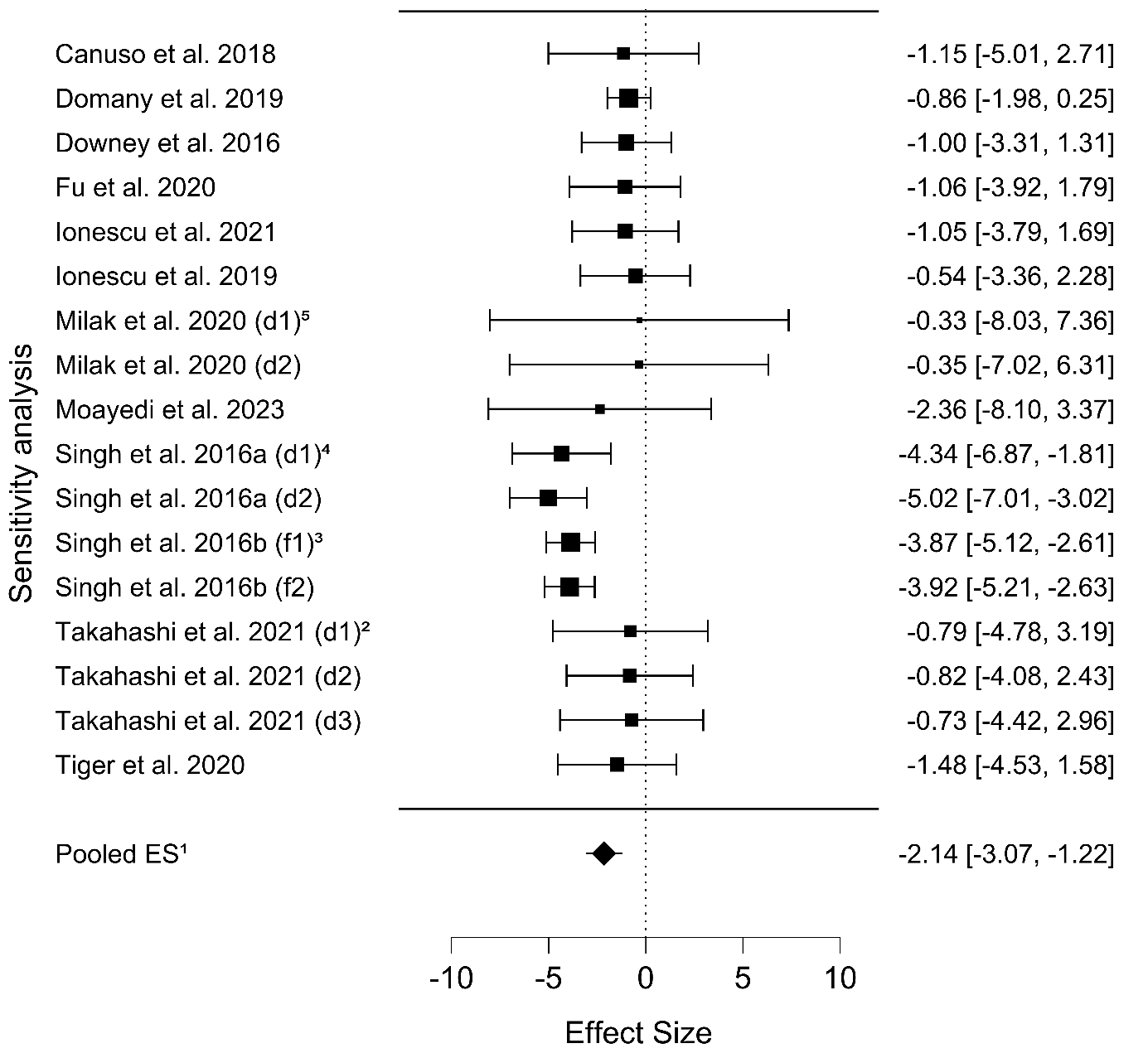


(1) ES = effect size (cohen‘s d)

(2) Takahashi et al. 2021: d1 = 28 mg esketamine ; d2 = 56 mg esketamine ; d3 = 84mg esketamine

(3) Singh et al. 2016b: f1 =  2 x week; f2 = 3 x week

(4) Singh et al. 2016a: d1 = 0,2 mg/kg esketamine d2 = 0,4 mg/kg esketamine

(5) Milak et al 2020: d1 = 0.4 mg/kg ketamine; d2 = 0.5 mg/kg ketamine

## eFigure 5: Placebo and treatment response 7 days post-intervention after sensitivity analysis

**eFigure 5 Part A: Forest plot after sensitivity analysis of placebo response 7 days post-intervention dpl7d(sens) = -1.08; z = -2.90; p = .004; [CI 95%, -1.80 to -0.35]; I² = 0 %**


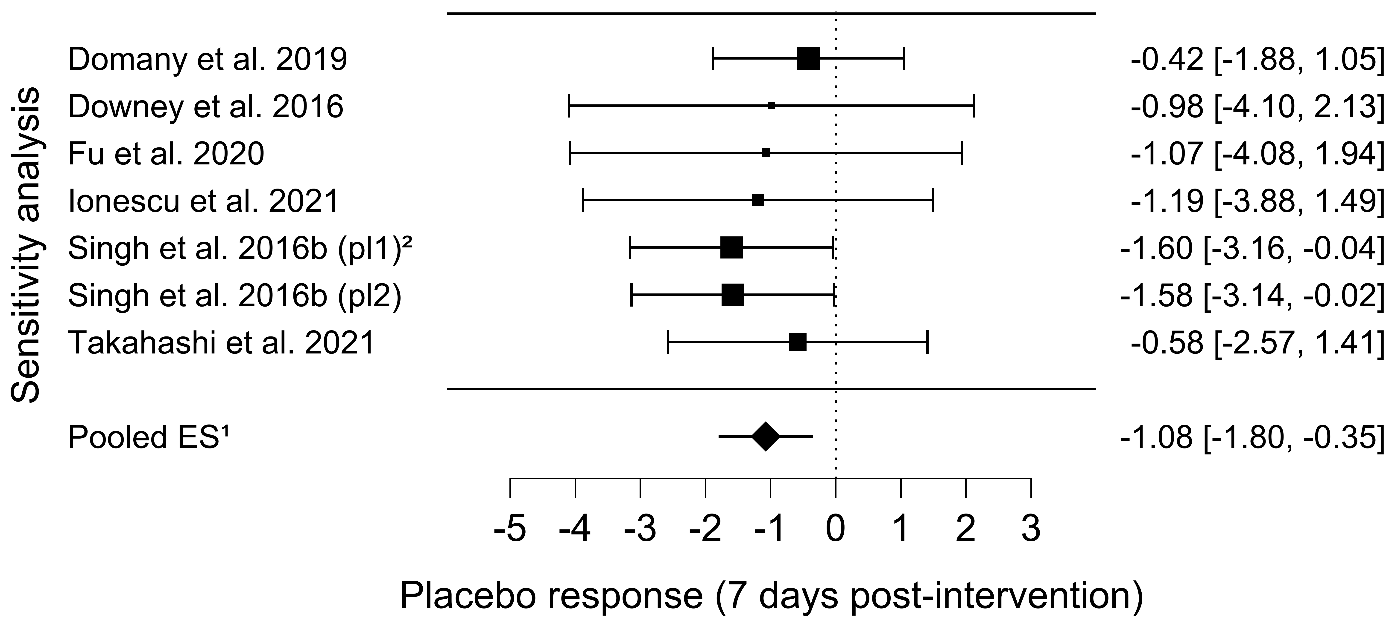


(1) ES = effect size (cohen‘s d); (2) Singh et al. 2016b: pl1 = first placebo group pl2 = second placebo group

**eFigure 5 Part B: Forest plot after sensitivity analysis of treatment response 7 days post-intervention dtr7d(sens) = -2.48; z = 3.08; p < .001; [CI 95% -4.06 to -0.90]; I² = 81,41 %**


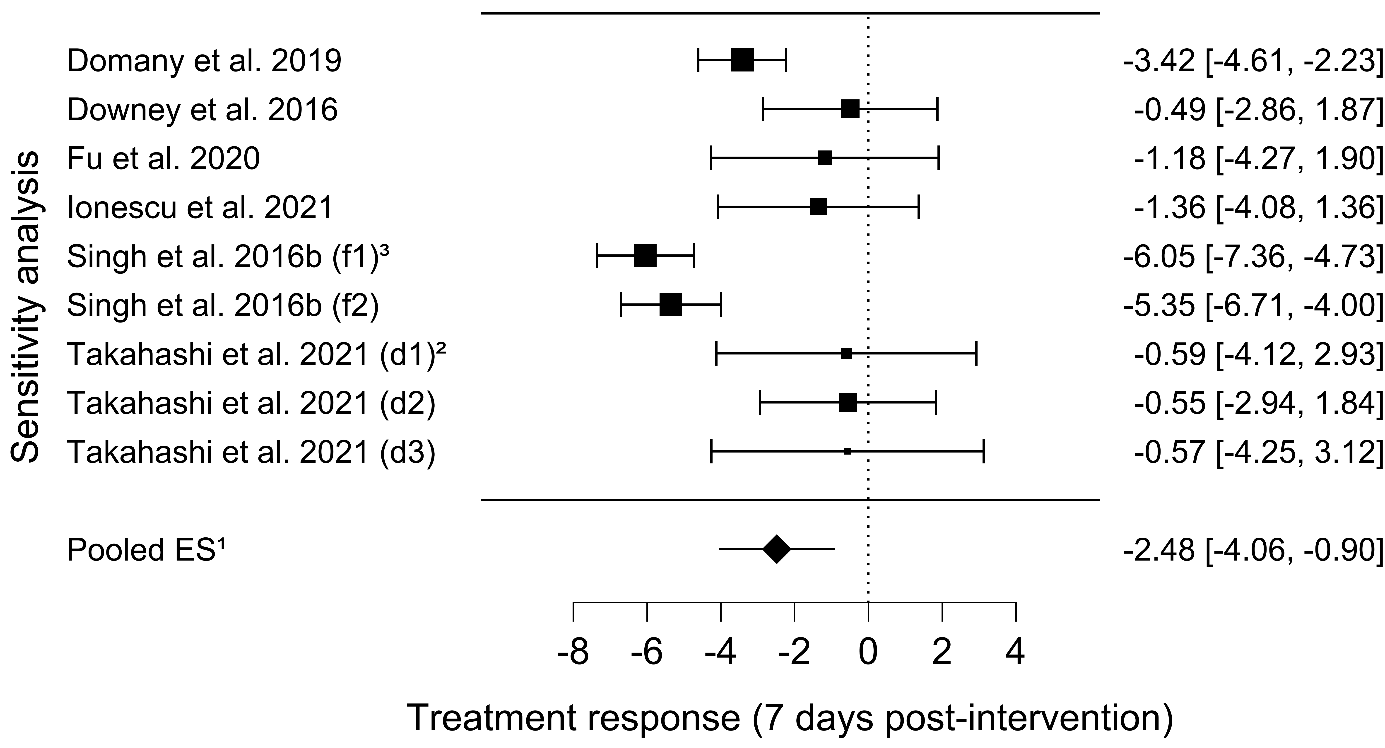


(1) ES = effect size (cohen‘s d); (2) Takahashi et al. 2021: d1 = 28 mg esketamine ; d2 = 56 mg esketamine ; d3 = 84mg esketamine; (3) Singh et al. 2016b: f1 =  2 x week; f2 = 3 x week

## eFigure 6: Rate of specific AEs to total AEs


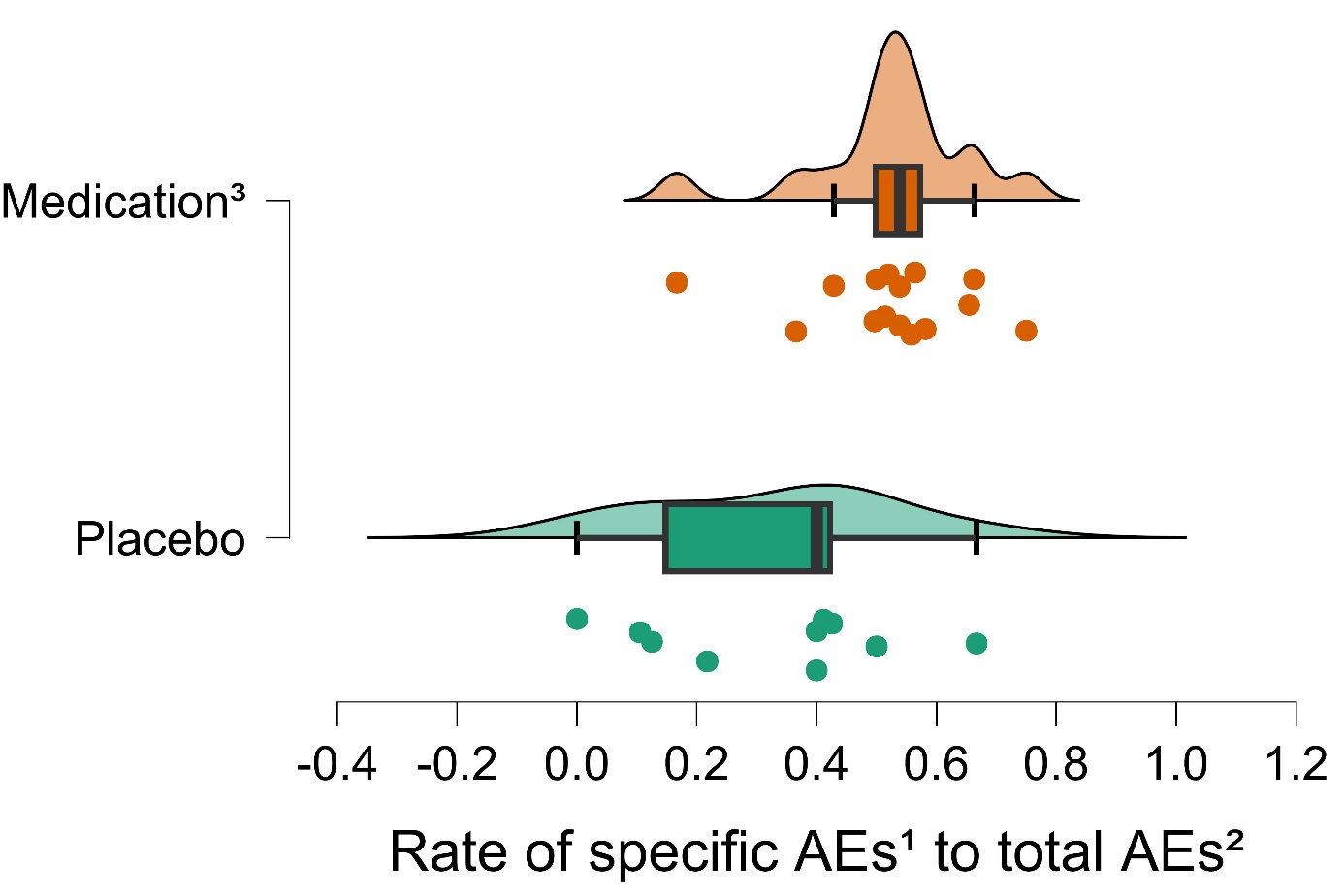


**Raincloud plot visualizing the results of the unpaired T-Test for the rate of specific AEs to total AEs between the medication and placebo groups.**

The rate specific AEs to total AEs is significantly higher than in the placebo group (14) = -2.67; p < .009.

(1) Specific adverse events (AEs) include medication-related AEs as described in the information leaflet for ketamine and esketamine

(2) Total AEs include the summary of all reported AEs

(3) Medication = ketamine and esketamine

REFERENCES

1. Pfizer Limited. *Ketalar® 10 mg/ml, 50 mg/ml and 100 mg/ml Injection. Ketamine hydrochloride.* (2016) [cited 2024 Jan 01].

2. Janssen Pharmaceuticals, Inc. *Spravato (Esketamine) nasal spray. Highlights of prescribing information* (2020) [cited 2024 Jan 01]. Available from: https://www.janssenlabels.com/package-insert/product-monograph/prescribing-information/SPRAVATO-pi.pdf
